# Microendoscopic Dorsal Laminectomy for Multi-Level Cervical Intervertebral Disc Protrusions in Dogs

**DOI:** 10.3390/vetsci9010018

**Published:** 2022-01-05

**Authors:** Hiroaki Kamishina, Yukiko Nakano, Kohei Nakata, Shintaro Kimura, Yuta Nozue, Adam G. Drury, Sadatoshi Maeda

**Affiliations:** 1 Joint Department of Veterinary Medicine, Faculty of Applied Biological Sciences, Gifu University, 1-1 Yanagido, Gifu 501-1193, Japan; sadat@gifu-u.ac.jp; 2 The United Graduate School of Veterinary Sciences, Gifu University, 1-1 Yanagido, Gifu 501-1193, Japan; shinta_ta_ta@yahoo.co.jp; 3 The Animal Medical Center, Faculty of Applied Biological Sciences, Gifu University, 1-1 Yanagido, Gifu 501-1193, Japan; ynakano@gifu-u.ac.jp (Y.N.); k.nakata.1986@gmail.com (K.N.); ytnz.23@gmail.com (Y.N.); 4 Virginia-Maryland College of Veterinary Medicine, Virginia Polytechnic Institute and State University, 205 Duck Pond Drive, Blacksburg, VA 24061, USA; druryvet@gmail.com

**Keywords:** endoscope, minimally invasive surgery, intervertebral disc protrusion, dog, cervical, dorsal laminectomy

## Abstract

The objective of this study was to evaluate the feasibility and clinical outcomes of microendoscopic dorsal laminectomy for multi-level cervical intervertebral disc protrusions in dogs. Eight client-owned dogs diagnosed with multi-level cervical intervertebral disc protrusions using computed tomography (CT) and magnetic resonance imaging (MRI) were included in this retrospective case series. Microendoscopic dorsal laminectomies (MEL) were performed with an integrated endoscopic system to the cranial and caudal vertebrae of the affected intervertebral joints. Pre- and post-operative neurological status, operation time, intra-operative complications, and postoperative complications were reviewed. Post-operative CT images were obtained to measure the dimensions of laminectomy and compared to those of planned laminectomy. Full endoscopic procedures were feasible in 7 dogs (87.5%) and the laminectomy dimensions were in agreement with pre-operative planning. In all dogs, major intra- and postoperative complications did not occur. Conversion to open surgery was required in one case. Short-term postoperative clinical deterioration was found in two dogs. Long-term clinical outcomes were good and comparable to those reported in previous studies of open dorsal laminectomies. MEL is a promising minimally invasive approach to multi-level cervical dorsal laminectomy for intervertebral disc protrusions. This technique may improve postoperative discomfort compared to the open approach. Further studies are needed to directly compare outcomes between these two approaches.

## 1. Introduction

Cervical myelopathy is a common neurological condition in dogs, which most often results from intervertebral disc herniation [1]. In cases with severe clinical signs, surgical treatment is indicated. The primary goal of surgical treatment for cervical intervertebral disc herniation is decompression of the spinal cord, which is achieved by either a ventral, dorsal, or dorsolateral approach [2]. The ventral approach, known as a ventral slot, is most often used but is usually applied to only one or two sites with material that is primarily ventral to the spinal cord. Dorsal laminectomy via a dorsal approach is selected when the disc material is located lateral or dorsal to the spinal cord or when spinal cord compression is present at multiple intervertebral disc levels [3,4,5]. Neurological deterioration is the major concern as a post-operative complication after dorsal laminectomy of the cervical vertebral column [6]. One reason that could contribute to prolonged recovery is that the extensive soft tissue disruption required to perform an open approach may result in significant postoperative discomfort. This prompted us to explore a less invasive approach to the dorsal cervical vertebral column to reduce the discomfort associated with the standard open approach.

In humans, minimally invasive surgeries are becoming more common. They were initially applied to disorders of the lumbar spine [7] and are becoming increasingly popular for various cervical spinal disorders. The advantages of minimally invasive surgery include smaller skin incisions, less muscle dissection and stripping, and reduced blood loss, leading to less post-operative pain, better mobility, and faster recovery [8,9,10,11]. Equivalent outcomes for lumbar disc prolapse between the standard microsurgical discectomy and a minimally invasive microscopic procedure were reported in a prospective randomized controlled study [12]. Compared to open foraminotomy, reduced post-operative pain and blood loss and equivalent clinical outcomes were reported in patients undergoing minimally invasive cervical foraminal decompression [13,14] and anterior cervical discectomy and fusion [15].

In the veterinary literature, most studies described a minimally invasive approach to the spine in canine cadavers or clinically normal dogs. Endoscope-assisted [16,17] and full-endoscopic procedures for the thoracolumbar spine have been reported [18,19,20]. The use of a minimally invasive device with video assistance was reported in canine cadavers and in 10 clinical cases that underwent ventral slot decompression [21]. The use of a minimally invasive expandable retractor to the lumbosacral region was reported in canine cadavers but this study did not use an endoscope for the visualization of the surgical field [22]. Wood et al. [23] reported endoscope-assisted lumbosacral foraminotomy in clinically normal dogs. Currently, there are no studies that describe a minimally invasive approach to the dorsal cervical vertebral column or clinical outcomes in dogs.

In the present study, we evaluated full-endoscopic dorsal laminectomy for multi-level cervical intervertebral disc protrusions in eight dogs.

## 2. Materials and Methods

### 2.1. Case Enrollment

This study was conducted as a descriptive retrospective clinical case series including client-owned dogs with multi-level cervical intervertebral disc protrusions. Imaging studies and surgical procedures were performed at the Animal Medical Center of Gifu University. All procedures were performed in accordance with the guidelines regulating animal use and ethics at Gifu University. Medical records of dogs that had undergone surgical treatment for multi-level cervical intervertebral disc protrusions using the microendoscopic technique were identified and included in the study. Informed consent of the owners was obtained prior to video recording of the gait (using an iPad Mini, Apple Inc., Cupertino, CA, USA), imaging studies, and surgery.

### 2.2. Imaging

In all dogs, anesthesia was induced by propofol at a dose of 5.0 mg/kg and maintained by sevoflurane and oxygen. The level of sevoflurane was set 1.3 times below the minimum alveolar concentration of sevoflurane and the end-tidal concentration was monitored. Magnetic resonance imaging (0.4 T, APERTO Eterna, Hitachi Healthcare, Tokyo, Japan; 3.0 T, Achieva dStream, Philips, Amsterdam, The Netherland) was performed to diagnose cervical intervertebral disc protrusion. Transverse and sagittal images of T2-weighted, T1-weighted, and fluid-attenuated inversion recovery images of the cervical spinal cord segment were acquired. Intervertebral disc protrusions (i.e., Hansen Type II) were diagnosed as previously reported [24]. Briefly, the affected intervertebral disc was classified as disc protrusion if either the disc protruded in a ventral or ventrolateral portion of the vertebral canal or if there was a symmetric uniform extension of the outer disc margin circumferentially that compressed the spinal cord. Computed tomography (CT) (Alexion Advance, Canon Medical System Corporation, Tochigi, Japan) with a slice thickness of 0.5–1.0 mm was used to obtain pre- and postoperative images of the cervical spinal cord.

### 2.3. Clinical Information and Follow-Up Evaluation

Clinical information of the cases regarding signalment, medical treatment received, locations of disc protrusions, and numbers of disc protrusions were recorded. The intra- and postoperative complications were reviewed from clinical records. Neurological grades at presentation, discharge, first hospital visit after surgery, and last hospital visit after surgery were recorded. In all cases, a telephone interview was performed in order to assess the current neurological status. Neurological grades were evaluated as previously reported by De Risio et al. [5]: Grade 0, normal neurologic status; Grade 1, ambulatory animal with a slightly reduced response to neurologic tests, typically manifested as mild ataxia and a slight delay in postural reaction testing; Grade 2, ambulatory animal with noticeable ataxia and paresis, with delayed postural reactions; Grade 3, ambulatory animal with paresis and absent postural reactions; Grade 4, a nonambulatory tetraparetic status with or without neck pain; Grade 5, tetraplegia and respiratory compromise. “Ambulation” was defined as the ability to walk 10 consecutive steps without support as previously reported [25].

### 2.4. Surgical Procedure

After premedication with atropine sulfate hydrate (0.01 mg/kg IV), midazolam (0.2 mg/kg IV), and ketamine hydrochloride (2 mg/kg IV), dogs were anesthetized using our standard protocol and maintained with sevoflurane in oxygen as described in the imaging study. Intra-operative analgesia for all study patients during surgery included constant rate infusion of fentanyl (3–15 μg/kg/h) and ketamine (0.12–0.6 mg/kg/h). Dogs were positioned in sternal recumbency on a vacuum beanbag with the neck slightly elevated and extended. The surgical area was prepared and draped including the area from the occipital bone to the spinous process of the 3rd thoracic vertebra.

**Figure 1:**
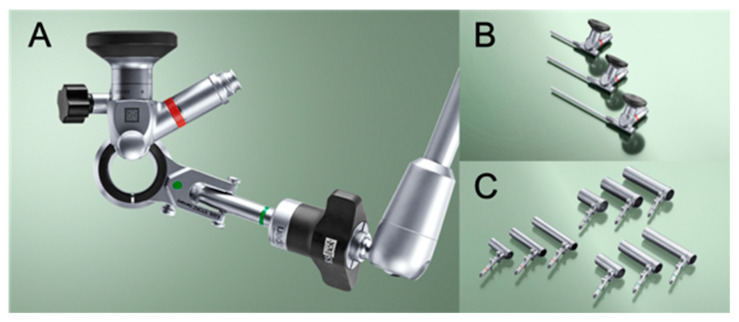
Images of the endoscopic system, the EasyGO! 2nd generation. (**A**), The integrated endoscopic system includes a tubular retractor, telescope, light source, and articulating locking arm. (**B**), The telescopes are forward-oblique 25° with an eyepiece angled at 90° and a diameter of 4 mm. Three different lengths are available: 60 mm, 90 mm, and 110 mm. (**C**), Three different outer diameters, 15 mm, 19 mm, and 23 mm of tubular retractors with different lengths, 40 mm, 45 mm, 70 mm, 75 mm, 90 mm, and 95 mm, compatible to short, middle, and long telescopes. Photos provided by and used with the permission of KARL STORZ Endoscopy Japan K.K.

All surgeries were performed with an integrated endoscopic surgical system (EasyGO! 2nd generation; KARL STORZ Endoscopy Japan K.K., Tokyo, Japan; Figure 1).

**Figure 2:**
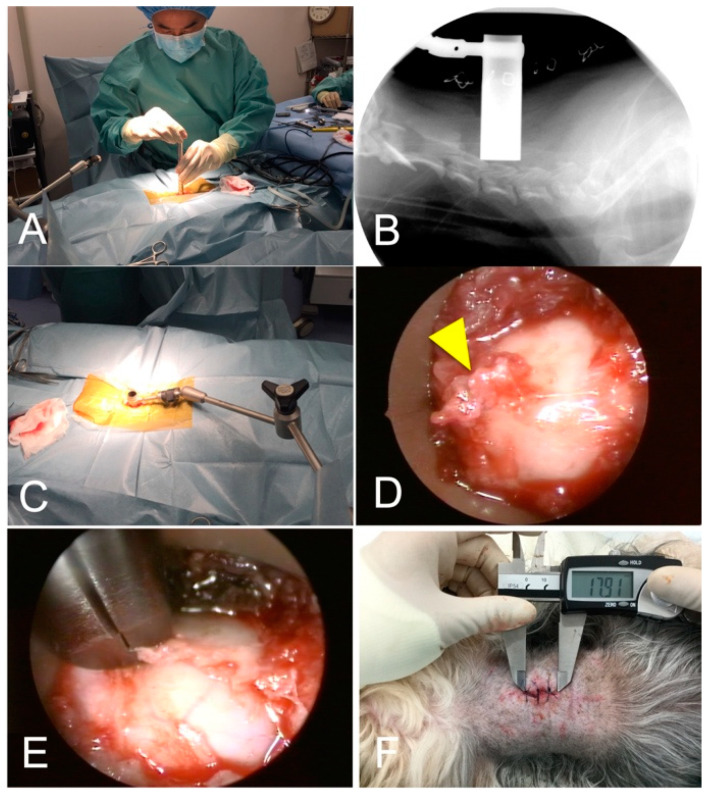
Procedures of microendoscopic cervical dorsal laminectomy (dog no. 6). (**A**), After the insertion of the smallest dilation tube through laterally divided ligamentum nuchae, muscle dilation was performed by serial placement of dilation tubes with increasing sizes. (**B**), The location of the tubular retractor was confirmed using fluoroscopy. (**C**), The tubular retractor was attached to an articulating locking arm that was clamped to the surgical table. (**D**), Epaxial muscles and soft tissues covering the vertebral arch were removed to expose the spinous process (arrowhead) and vertebral arch. (**E**), The vertebral arch was removed by a high-speed drill and Kerrison rongeurs. The cranial and caudal vertebral arches of the target location were removed according to the planned region of laminectomy. (**F**), After all laminectomies, the whole system was pulled back and removed from the surgical site. The skin closure was performed with two to three stitches of nylon. The cranial side of the dog is to the left in all images.

To maintain consistency, all surgical procedures were performed by the same individual (HK). A 2–3-cm skin incision was made over the dorsal midline of the neck with a No. 10 scalpel blade. Using gross visualization, the ligamentum nuchae was split along the midline. A small dilation tube (outer diameter 5.2 mm) was inserted to the level of the target vertebral arch. The location of the dilation tube was confirmed by a lateral view of fluoroscopy (ARCADIS Orbic, Siemens Healthcare, Erlangen, Germany). Serial dilation sleeves were placed over the smallest sleeve in increasing size to widen the port of entry (Figure 2A). A tubular retractor with an outer diameter of 15 mm or 19 mm, depending on the size of the dog, was placed over the dilation sleeves and positioned over the vertebral arch. The location of the sleeves was rechecked using fluoroscopy (Figure 2B). The tubular retractor was attached to an articulating locking arm that was clamped to the surgical table, followed by the removal of all dilation sleeves (Figure 2C). The telescope was attached to the telescope holder, and the light cable and camera head were connected. The light source (Cold Light Fountain XENON 300; KARL STORZ Endoscopy Japan K.K., Tokyo, Japan) was connected to provide a view of the surgical field on the monitor (Xenon Nova 300; KARL STORZ Endoscopy Japan K.K., Tokyo, Japan).

Epaxial muscles and soft tissues covering the vertebral arch were removed using spoon forceps, electrocautery, and a periosteal elevator (Figure 2D). The spinous process and vertebral arch were removed using a high-speed drill with a 2-mm diamond burr with frequent lavage. After the vertebral canal was entered, epidural fat was visualized, and Kerrison rongeurs (YDM CORPORATION, Tokyo, Japan) were used to complete the dorsal laminectomy (Figure 2E). The lateral extent of laminectomy was restricted to the inner boundary of the cranial and caudal articular processes. The craniocaudal extent of laminectomy was determined based on the location of disc protrusions. After completion of the first dorsal laminectomy, the tubular retractor was moved in situ to expose the vertebral arch of the next surgery site, and the dorsal laminectomy procedure was repeated. Because the size of the dorsal space between vertebral arches varies, a decision was made about the range of bone removal for each case, using pre-operative CT images. After all laminectomies, the entire system was removed from the surgical site. As in MEL in humans, suturing of muscle layers and subcutaneous tissues was not performed in this study. This is because paraspinal muscles usually come back together to their original position after removing the tubular retractor. For the same reason, a drainage tube was not placed. Skin closure was performed with two to three stitches of nylon (Figure 2F).

### 2.5. Post-Operative Management

For postoperative management of all cases, monitoring of neurological status was performed during hospitalization. Non-ambulatory dogs were rotated frequently to avoid decubitus ulcers. Postoperative analgesia during hospitalization was provided by constant rate infusion of fentanyl (1–2 μg/kg/h) and ketamine (0.04–0.08 mg/kg/h). Post-discharge recommendations included avoidance of stairs and the start of short walks on a harness for 2 weeks with a gradual increase in exercise over the next 4 to 6 weeks. Basic physiotherapy, including standing proprioceptive feedback and passive range of motion exercises, was also performed by owners. For nonambulatory tetraparetic cases, intensive nursing care and in-house physical therapy consisting of passive range of motion and cart-supported exercise were performed.

### 2.6. Imaging Analyses

Pre- and post-operative CT images were exported in the Digital Imaging and Communication in Medicine format to a commercial imaging software (OsiriX MD v.9.0, Pixmeo SARL, Bernex, Switzerland) for imaging analyses. The cervical vertebral column was scanned in 0.5–1.0 mm slices and the area of the planned laminectomy was measured. The planned width of laminectomy was defined as the width between the inner borders of the base of the articular processes. The cranial and caudal borders of laminectomy were half of the length of the vertebral arch of the most cranial or caudal vertebra of the target location. Post-operative CT imaging was performed immediately after surgery. On post-operative CT, the area of the laminectomy window was measured by manually tracing the border of the bone defect including the dorsal space between laminae. The ratio of bone removal compared to the planned area of laminectomy was calculated.

## 3. Results

### 3.1. Case Population

**Figure 3:**
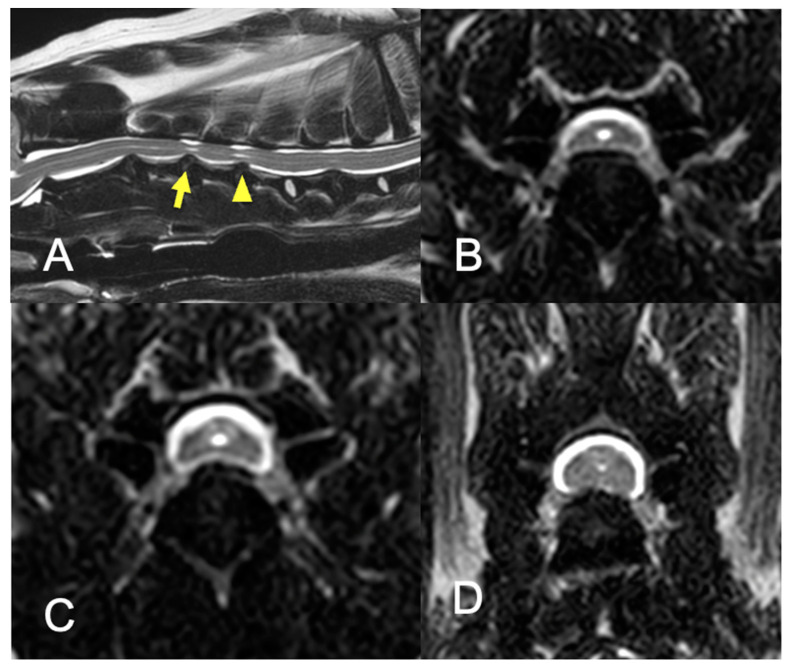
MRI findings of dog no. 6. (**A**), Sagittal T2-weighted image of the cervical spinal cord. The spinal cord is compressed at C3–4 (arrow) and C4–5 (arrowhead). The central canal is slightly dilated at the level of compression of both sites. Signal intensities of the nucleus pulposes are partially decreased at these intervertebral discs and intervertebral disc herniations are contained within the boundaries of the intervertebral disc space, supporting the diagnosis of intervertebral disc protrusion. (**B**), Transverse T2-weighted image of the spinal cord at the C3–4 intervertebral disc level. The spinal cord is ventrally compressed by a hypointense bulging structure continuous to the annulus fibrosus. (**C**), Transverse T2-weighted image of the spinal cord at the C4–5 intervertebral disc level. The spinal cord is ventrally compressed but the degree of spinal cord deformity is milder than C3–4. (**D**), Transverse T2-weighted image of the spinal cord at the C6–7 intervertebral disc level. Although the spinal cord is slightly deformed by the dorsally herniated anulus fibrosus, this location was judged clinically irrelevant because of the absence of intraparenchymal hyperintensity change.

Eight dogs with multi-level cervical intervertebral disc protrusions were included in this study (Figure 3). The breeds of dogs were: Chihuahua (*n* = 2), Yorkshire Terrier (*n* = 2), Whippet (*n* = 1), Toy Poodle (*n* = 1), Pomeranian (*n* = 1), and Doberman Pinscher (*n* = 1). Four dogs were male, two of which were castrated, and four dogs were female, three of which were spayed. The median body weight was 3.78 kg (range, 2.62–30.4 kg). The median age at onset was 117 months (range, 73–142.3 months), while that at surgery was 121.0 months (range, 84–143 months). Low-dose prednisolone was given to five dogs prior to surgery but discontinued after surgery. The number of intervertebral disc protrusions was three sites (*n* = 5), and two sites (*n* = 3). The median duration of clinical signs was 30 days (range, 14–330 days). Clinical information and the imaging findings of all cases are summarized in Table 1.

### 3.2. Surgery and Intraoperative Complications

Dorsal laminectomies were performed with a 19 × 70-mm tubular retractor (diameter and length) in one dog, with a 19 × 40-mm tubular retractor in one dog, and with a 15 × 40-mm tubular retractor in six dogs. The mean ± SD surgery time of all dogs was 181.6 ± 75.2 min. The surgical time was significantly protracted in two dogs (dog no. 4 and no. 8). In dog no. 4, the area of bone removal was found to be too small by postoperative CT, after which a revision open surgery was performed to extend the laminectomy under a surgical microscope. In dog no. 8, ventral slot procedure and interbody fusion cage placement were performed to the C3–4 intervertebral disc protrusion prior to endoscopic dorsal laminectomy. Excluding these two dogs, the mean ± SD surgery time was 148.8 ± 44.5 min. In all dogs, major intraoperative complications did not occur. In seven dogs that underwent MEL (excluding dog no. 4) the mean ± SD size of skin incision was 25.4 ± 5.9 mm. Data regarding surgeries are summarized in Table 2.

### 3.3. Postoperative Complications and Clinical Outcomes

At presentation, the neurological grades were grade 2 (dog no. 2), grade 3 (dog no. 1, 3, 4, 7), and grade 4 (dog no. 5, 6, 8). The median initial neurological grade at presentation was 3 (range, 2–4). Dog no. 4 was excluded from analyses of post-operative complications and outcomes because this dog received additional open surgery. In the perioperative follow-up period assessed on the day of discharge (median post-operative day 3, range 2–4), the neurological grades remained at the same grades as their pre-operative grades in six dogs (dog no. 1, 2, 3, 5, 6, 8). Although there were no serious postoperative complications, such as post-operative bleeding or severe pain, the neurological grades were worsened from grade 3 to 4 in one dog (dog no. 7) at discharge. Six dogs (dog no. 1, 2, 3, 6, 7, 8) were evaluated at the first recheck (median post-operative day 17, range 10–42). At the first recheck, the neurological grades were improved in five dogs (dog no. 1, 2, 3, 7, 8) from their neurological grades at discharge. One dog (dog no. 6) remained in the same grade as its initial grade (grade 4) at both discharge and first recheck (post-operative day 10), but improved to grade 1 at its last recheck (postoperative day 88). The latest assessment was performed by telephone interview with the owners. The median postoperative day of telephone interview was day 521 (range, 88–1024). The neurological grades were further improved in five dogs (dog no. 1, 2, 5, 7, 8) from their last evaluation and remained at the same grade in two dogs (dog no. 3, 6). The median neurological grades obtained by telephone interview was 1 (range, 0–1). Data of individual dogs are presented in Table 3.

### 3.4. Morphometric Analyses

**Figure 4:**
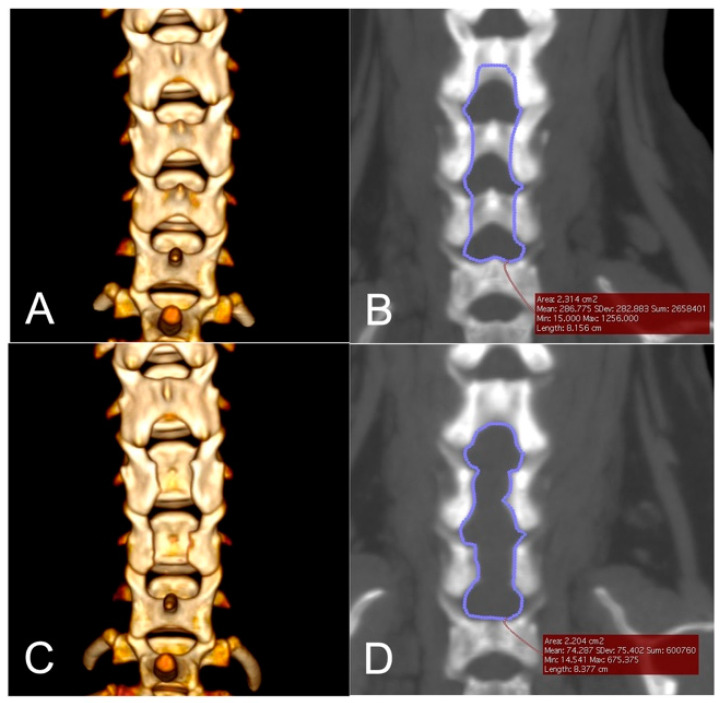
Morphometric analyses of the planned and post-operative laminectomy area of a dog undergoing the endoscopic procedure (dog no. 2). (**A**), A pre-operative dorsal view of 3-D volume rendering of the cervical vertebrae. The dog had intervertebral disc protrusions at C4–5, C5–6, and C6–7. (**B**), The planned area of dorsal laminectomy shown as a traced area was 2.314 cm^2^. Typically, the dorsal space between C6 and C7 is wide in small breed dogs and additional removal of the C7 vertebral arch was not performed. (**C**), A post-operative dorsal view of 3-D volume rendering of the cervical vertebrae. (**D**), The actual laminectomy area shown as a traced area was 2.204 cm^2^, resulting in an actual/planned laminectomy ratio of 95.25%.

**Figure 5:**
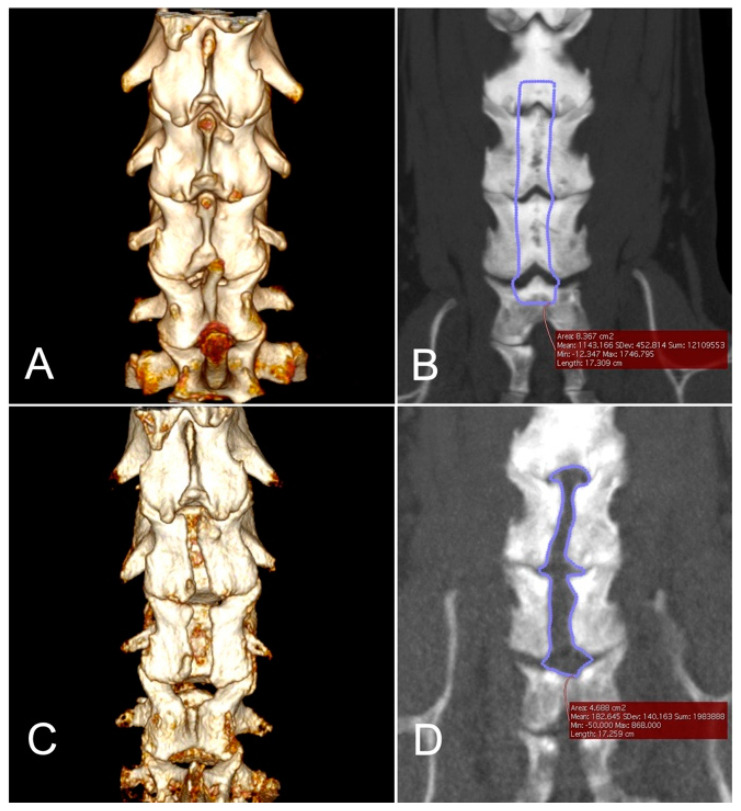
Morphometric analyses of the planned and post-operative laminectomy area of a dog that received a revision open laminectomy (dog no. 4). (**A**), A pre-operative dorsal view of 3-D volume rendering of the cervical vertebrae. This dog had intervertebral disc protrusions at C4–5, C5–6, and C6–7. (**B**), The planned area of dorsal laminectomy shown as a traced area was 8.367 cm^2^. (**C**), A post-operative dorsal view of 3-D volume rendering of the cervical vertebrae prior to a revision open laminectomy. (**D**), The actual laminectomy area shown as a traced area was 4.688 cm^2^, resulting in an actual/planned laminectomy ratio of 56.03%.

Laminectomy dimensions were measured on pre-operative and postoperative CT images. Post-operative CT was not performed in one dog (dog no. 5) due to persistent hypotension during surgery. The mean ± SD of the planned laminectomy area was 3.07 ± 2.43 cm^2^, and the mean ± SD of the actual laminectomy area was 2.38 ± 1.09 cm^2^. The mean ± SD ratio of the actual laminectomy area to the planned laminectomy area was 88.57 ± 18.89%. The actual/planned area of laminectomy was the smallest in dog no. 4 (56.03%), which underwent a revision open surgery. Representative images of dogs that underwent the endoscopic procedure (dog no. 2) and that underwent a revision open dorsal laminectomy (dog no. 4) are illustrated in Figure 4 and Figure 5, respectively.

## 4. Discussion

This case series suggests that MEL is a feasible surgical procedure for multi-level cervical intervertebral disc protrusions in dogs. There were no major intraoperative complications and conversion to an open approach was required in only one case due to inadequate decompression. The clinical signs improved at either the short-term (*n* = 6) or long-term (*n* = 2) postoperative period. Although clinical deterioration in the perioperative period was noted in two out of eight dogs (25%), the clinical signs improved over the long term in all dogs. These findings suggest that MEL is a potential alternative to conventional open procedures in dogs with multi-level cervical intervertebral disc protrusions, offering the advantages of minimally invasive intervention.

For cervical disc diseases in dogs, the ventral slot procedure is the most frequently described surgical technique [2]. However, there are limitations to the size and number of ventral bony windows that can be safely made to avoid compromising the stability of the vertebrae. The ventral slot procedure has been documented to cause instability of the caudal cervical vertebrae [26,27,28]. Dorsal laminectomies may be more effective when intervertebral disc protrusions are present at multiple levels, especially in the caudal cervical regions that frequently represent Hansen type-2 disc disease. In our study, all of the cases had multi-level disc protrusions and five out of eight dogs had more than three-disc protrusions involving the caudal cervical vertebrae. In addition, cases included in this study were middle-aged or old dogs. As multi-level intervertebral disc protrusions in the caudal cervical regions are likely associated with age-related changes of the intervertebral structures, the incidence of disc diseases in these locations is higher than previously recognized [27,29].

The clinical outcomes of the dogs in this study were considered satisfactory as the neurological grades of all dogs improved after surgery. Cervical dorsal laminectomy via an open approach has been reported in previous studies with clinical improvement in most cases long term [3,5]. In a report of 20 dogs with caudal cervical spondylomyelopathy (CCSM) undergoing dorsal laminectomy, 19 dogs improved but the mean time to optimal recovery was as long as 3.6 months post-surgery [5]. In small breed dogs <15 kg with cervical intervertebral disc diseases, cervical dorsal laminectomies were performed and 67% recovered normal ambulation 2 weeks post-operatively and 100% were ambulatory at final recheck 5–44 months postoperatively [3]. In our study, among dogs who underwent full-endoscopic surgery, five out of six dogs achieved neurological improvement at the first recheck post-operative evaluation (median postoperative day 17), suggesting dorsal laminectomy by the endoscopic procedure may lead to faster recovery than an open dorsal laminectomy. In humans, clinical recovery rates have been compared between minimally invasive spinal surgeries and open surgeries, and it seems to be superior with minimally invasive spinal surgeries in the short term; however, whether the benefits can be maintained long term remains unclear [8,10,11]. The current study is the first to report the feasibility and short-term clinical benefits of MEL in dogs, although the safety and long-term clinical outcomes need to be evaluated in future studies.

Previous reports on the incidence of postoperative complications in dogs receiving dorsal decompression surgeries have been controversial [3,4,5,30,31,32]. The increased risk of postoperative morbidity, complications, prolonged hospital stay, and delayed functional recovery have been reported in dogs with CCSM receiving dorsal laminectomy via an open approach [5,30,31]. Of postoperative complications, clinical worsening is the major concern after cervical dorsal laminectomy. In one study, 70% of the dogs with CCSM undergoing cervical dorsal laminectomy had clinical worsening during the early postoperative period [5]. In our study population, two out of eight dogs showed clinical deterioration during the perioperative period, but these two dogs returned to their original neurologic status at the first recheck in-hospital evaluation, on postoperative day 15 and day 16, respectively. Therefore, MEL may be advantageous over dorsal decompression via an open approach as it may decrease the risk of postoperative morbidity and enhance faster recovery. In humans, the reported benefits of minimally invasive spinal surgeries are multiple, including decreased soft tissue trauma, decreased scar tissue formation, less bleeding, decreased operating times, decreased infection rate, decreased immediate postoperative and long-term pain, and faster return to function [8,9,10,11]. Decreasing tissue dissection and preserving muscles, and tendinous and ligamentous attachments should increase perispinal stability, decreasing the risk of post-operative complications such as vertebral luxation. Whether these advantages of minimally invasive surgeries reported in humans apply to dogs with cervical disc diseases warrants further investigations. A recent study in research dogs looking into serum creatine kinase level evaluation and mechanical sensory thresholds after the minimally invasive approach to the thoracolumbar spinal cord demonstrated reduced muscle damage and post-operative pain benefits [33].

Similar to open dorsal laminectomy, direct access and removal of disc materials located ventrally or ventrolaterally to the spinal cord is impossible with MEL. Therefore, this technique is not indicated for a single-level acute extrusion of the intervertebral disc material. Although we have not performed MEL for dogs with laterally herniated disc material, it has been successfully treated by endoscopic posterior foraminotomy in humans [13,15]. In addition, as in other endoscopic surgeries, there is a steep learning curve inherent to this surgical procedure [34,35]. The steep learning curve may arise from two factors: the diminished perception of depth and the restricted working space. Compared to microscopic surgeries, the perception of depth is diminished in endoscopic surgery, which is possibly associated with an increased risk of iatrogenic injury to the nervous tissues [36,37]. While limiting damage to surrounding soft tissues, the tubular system restricts the working space and complicates the manipulation of surgical instruments which may result in a lower chance of identifying and removing free fragments of disc materials [36,37,38]. We had to convert to open surgery in one dog because the laminectomy area was considered too small based on postoperative CT images. The main reason for not being able to extend the laminectomy was because of limited mobility of the tubular system to allow wide bone removal. This problem may arise in large dog breeds with larger paraspinal muscles and deep surgical fields that limit the mobility of the tubular system.

There were several limitations in the present study including the small sample size, short in-hospital observation period, and lack of direct comparison to other surgical techniques. Long-term neurological grading may have been inaccurate because the final evaluation was based on a telephone interview of the owners. The size of the dogs was skewed to small breed dogs reflecting the general dog population in Japan; however, this may not be representative of the clinical population of other countries. The minimally invasive nature of this technique was not directly assessed in this study. In particular, the amount of soft tissue damage, post-operative pain, rate of complication, and recurrence should be investigated in a future prospective study. The effects of the learning curve on clinical outcomes should also be investigated in order to expand this surgical technique to the general clinical population.

## 5. Conclusions

Full endoscopic procedures were feasible for multi-level cervical intervertebral disc protrusions in this small case series. The actual laminectomy dimensions were in agreement with pre-operative planning in six out of seven dogs that received morphometric analysis. One dog received additional open surgery due to inadequate bone removal. Short-term postoperative clinical deterioration was noted in two dogs, but long-term clinical outcomes were good and comparable to those reported in previous studies of open dorsal laminectomy. The safety, effects of the learning curve, long-term clinical outcomes, and minimally invasive nature of this procedure need to be investigated in future prospective studies. Alongside technical factors, the cost of the procedure needs to be considered from the perspectives of both veterinarians and owners.

## Figures and Tables

**Table 1:** Clinical information of the dogs included in this study. M, male; CM, castrated male; F, female; SF spayed female.

Case No.	Breed	Body Weight at Surgery (kg)	Sex	Age at Surgery (month)	Duration of Clinical Signs(days)	Locations of IVDH	Prior Medication
1	Chihuahua	3.2	CM	84	180	C4–5, C5–6, C6–7	prednisolone 0.3 mg/kg q24
2	Chihuahua	2.62	CM	124	14	C4–5, C5–6, C6–7	none
3	Whippet	11.6	M	118	120	C5–6, C6–7	none
4	Doberman Pinscher	30.4	SF	108	21	C4–5, C5–6, C6–7	prednisolone 0.5 mg/kg q24
5	Yorkshire Terrier	3.55	CM	143	330	C5–6, C7–T1	prednisolone 0.5 mg/kg q24
6	Yorkshire Terrier	4	SF	124	14	C3–4, C4–5	prednisolone 0.6 mg/kg q48
7	Toy Poodle	4.3	F	142	30	C5–6, C6–7, C7–T1	prednisolone 0.5 mg/kg q24
8	Pomeranian	2.9	SF	118	30	C3–4, C6–7, C7–T1	none

**Table 2:** Locations of dorsal laminectomy, surgery time, size of tubular retractor, size of skin incision, and dimensions of planned and actual laminectomy. NA, not available. * The surgery time includes post-operative CT and a revision open laminectomy. ** The surgery time includes interbody fusion cage placement in the C3–4 disc space.

Case No.	Locations of Laminectomy	Total Surgery Time (min)	Size of Tubular Retractor (mm)	Size of Skin Incision (mm)	Laminectomy Size		
					Planned Area (cm^2^)	Actual Area (cm^2^)	Actual/Planned (%)
1	142	15 × 40	26.19	2.176	2.002	92.00
2	C4, C5, C6	125	15 × 40	26.5	2.314	2.204	95.25
3	C5, C6, C7	189	19 × 40	19.1	3.552	2.682	75.51
4	C4, C5, C6, C7	324 *	19 × 70	NA	8.367	4.688	56.03
5	C5, C6, C7	113	15 × 40	30.24	NA	NA	NA
6	C3, C4, C5	217	15 × 40	17.91	1.559	1.374	88.13
7	C5, C6, C7	107	15 × 40	34.62	1.993	1.919	96.29
8	C6, C7	236 **	15 × 40	23.4	1.519	1.774	116.79

**Table 3:** Neurological grades of eight dogs at different time points. NA, not available.

Case No.	Neurological Grade (Post-Op Day)					In-Hospital Follow Up Evaluation (Days)
	Presentation	Discharge	1st Visit Post-Surgery	Last Visit Post-Surgery	Telephone Interview	
1	3	3 (2)	2 (42)	NA	0 (898)	42
2	2	2 (3)	1 (17)	NA	0 (1024)	17
3	3	3 (3)	1 (25)	NA	1 (437)	25
4	3	4 (3)	2 (15)	1 (92)	1 (284)	15, 43, 92
5	4	4 (3)	NA	NA	1 (668)	NA
6	4	4 (2)	4 (10)	1 (88)	1 (521)	10, 22, 63, 88
7	3	4 (4)	2 (16)	NA	1 (495)	16
8	4	4 (3)	2 (17)	NA	1 (88)	17

## Data Availability

All data sets obtained and analyzed during the experiment are available upon reasonable request from the respective author.
